# Comparing No-Code Platforms and Deep Learning Models for Glaucoma Detection From Fundus Images

**DOI:** 10.7759/cureus.81064

**Published:** 2025-03-24

**Authors:** Mauro Gobira, Luis F Nakayama, Caio Vinicius S Regatieri, Rubens Belfort

**Affiliations:** 1 Ophthalmology, Vision Institute - Instituto Paulista de Estudos e Pesquisas em Oftalmologia (IPEPO), São Paulo, BRA; 2 Ophthalmology, Universidade Federal de São Paulo (UNIFESP), São Paulo, BRA

**Keywords:** acrima dataset, create ml, deep learning, fundus images, glaucoma detection, resnet200d, teachable machine

## Abstract

Purpose: This study compares the performance of two no-code machine learning platforms, Google's Teachable Machine (TM) (Google LLC, Mountain View, CA, USA) and Apple's Create ML (Apple Inc., Cupertino, CA, USA), alongside a traditional deep learning model, ResNet200d, in classifying optic nerve fundus images into glaucoma and non-glaucoma categories using the ACRIMA dataset.

Methods: A comparative cross-sectional analysis was conducted using 705 labeled fundus images from the ACRIMA dataset (326 glaucomatous, 239 non-glaucomatous). Models were trained separately on each platform, and a validation set comprising 70 glaucomatous and 70 non-glaucomatous images was used to assess performance. Performance metrics, such as sensitivity, specificity, F1 score, and Cohen's kappa, were assessed with 95% confidence intervals. Statistical analysis was performed using DATAtab (DATAtab e.U. Graz, Austria (https://datatab.net)).

Results: The ResNet200d model demonstrated the highest performance, with an accuracy of 99.29%, a sensitivity of 98.57%, a specificity of 100%, and an F1 score of 99.29%. Create ML achieved a sensitivity of 93.24%, a specificity of 98.48%, and an F1 score of 95.83%. TM exhibited a sensitivity of 95.71%, a specificity of 94.29%, and an F1 score of 95.04%. Both no-code platforms demonstrated strong performance, with Create ML excelling in specificity and TM showing higher sensitivity.

Conclusion: While the ResNet200d model outperformed both no-code platforms in diagnostic accuracy, the no-code platforms demonstrated robust capabilities, highlighting their potential to democratize artificial intelligence (AI) in healthcare. These results highlight the potential of no-code platforms for democratizing medical image analysis, especially in resource-limited contexts. Further studies with diverse datasets are recommended to validate these results.

## Introduction

No-code platforms are gaining popularity by enabling non-programmers to efficiently develop applications and systems [1]. These platforms, widely discussed in the context of machine learning and data management, use intuitive interfaces and pre-built components to streamline application development. For example, no-code machine learning platforms allow users to perform tasks such as time series forecasting without requiring programming skills, although many are not free. Similarly, low-code/no-code platforms enhance business agility and democratize application development but may present challenges, such as security and data governance issues [2]. 

The advancement of artificial intelligence (AI) in ophthalmology has been marked by significant progress in diagnostic imaging, surgical applications, and patient management [2,3]. Deep learning and machine learning technologies have the potential to improve ophthalmic care by increasing the diagnostic accuracy of diseases such as diabetic retinopathy, age-related macular degeneration, and glaucoma through automated retinal image analysis. In glaucoma diagnostics, convolutional neural network (CNN) architectures such as VGG-19, ResNet, MobileNet, and Inception have been extensively studied and adopted at various levels [4,5]. These models excel in feature extraction and classification tasks, enabling the robust identification of glaucomatous changes in fundus and optical coherence tomography (OCT) images. The increasing adoption of these technologies and ongoing research highlight AI's potential to enhance clinical workflows and improve patient outcomes in glaucoma care. 

In this evolving landscape, several no-code platforms have been developed to facilitate machine learning model creation, including Amazon Web Services (Amazon.com, Inc., Seattle, WA, USA), Google Cloud AutoML (Google LLC, Mountain View, CA, USA), Microsoft Azure ML (Microsoft Corporation, Redmond, WA, USA), Apple's Create ML (Apple Inc., Cupertino, CA, USA), and MedicMind (MedicMind, London, UK) [6]. These platforms enable healthcare professionals to develop diagnostic models without requiring advanced technical expertise. Some offer free versions or limited free features, while others require a subscription or a one-time payment. However, despite their growing potential and popularity, there is still a lack of systematic research evaluating the accuracy and reliability of models developed using these tools in ophthalmology, particularly in disease diagnosis based on retinal images. To address this gap, this study aims to assess and compare the performance of two widely used no-code machine learning platforms, Google's Teachable Machine (TM) (Google LLC, Mountain View, CA, USA) and Apple's Create ML, alongside the traditional ResNet200d model, in classifying optic nerve fundus images into glaucoma and non-glaucoma categories.

## Materials and methods

Study design and ethical considerations 

This study employed a comparative cross-sectional design to evaluate the classification performance of three image recognition models: ResNet200d, Google's TM, and Apple's Create ML. These models were trained and tested on optic nerve fundus images to distinguish between glaucomatous and non-glaucomatous cases. The ACRIMA dataset, comprising 705 anonymized and pre-labeled optic nerve fundus images, was utilized for model development and evaluation. Ethical approval was not required under Brazilian Resolution CNS No. 510/2016, as the dataset is publicly available and does not contain personally identifiable information. The study adhered to the ethical principles outlined in the Declaration of Helsinki.

Dataset description and preprocessing 

The ACRIMA dataset, utilized exclusively for classification tasks in this study, comprises optic nerve fundus images captured using the Topcon TRC retinal camera (Topcon Corporation, Tokyo, Japan) paired with the IMAGEnet® capture system, which features a 35° field of view [7]. Before capturing these images, pupil dilation was performed, and each image was meticulously centered on the optic disc. To ensure high-quality data, any images with artifacts, noise, or poor contrast were systematically excluded from the dataset. The labeling of the images was meticulously carried out by two independent glaucoma experts, each with eight years of experience, without the aid of additional clinical information. Notably, the dataset provided only images for classification purposes and did not include segmentation data for the optic disc or optic cup. The dataset can be publicly accessed on Kaggle (Kaggle, San Francisco, CA, USA) at https://www.kaggle.com/datasets/toaharahmanratul/acrima-dataset.

Model development, training, and testing

For the development of all three models (ResNet200d, Google's TM, and Apple's Create ML), the dataset was divided into 80% for training (565 images) and 20% for testing (140 images). Each model was trained and tested using the same dataset to ensure consistency in evaluation.

The ResNet200d CNN, pre-trained on ImageNet, was implemented using PyTorch 1.12.1. Retinal images were resized to 224 × 224 pixels and normalized based on ImageNet preprocessing standards. The training was conducted over 16 epochs, with a batch size of 29, using the Adam optimizer. The initial learning rate was set to 0.001 and dynamically reduced to 1.0000e-05 when validation loss plateaued. An early stopping criterion with a patience of 10 epochs was applied to prevent overfitting. Model checkpoints were saved at each epoch, and the best-performing model was selected based on the lowest validation loss.

**Figure 1:**
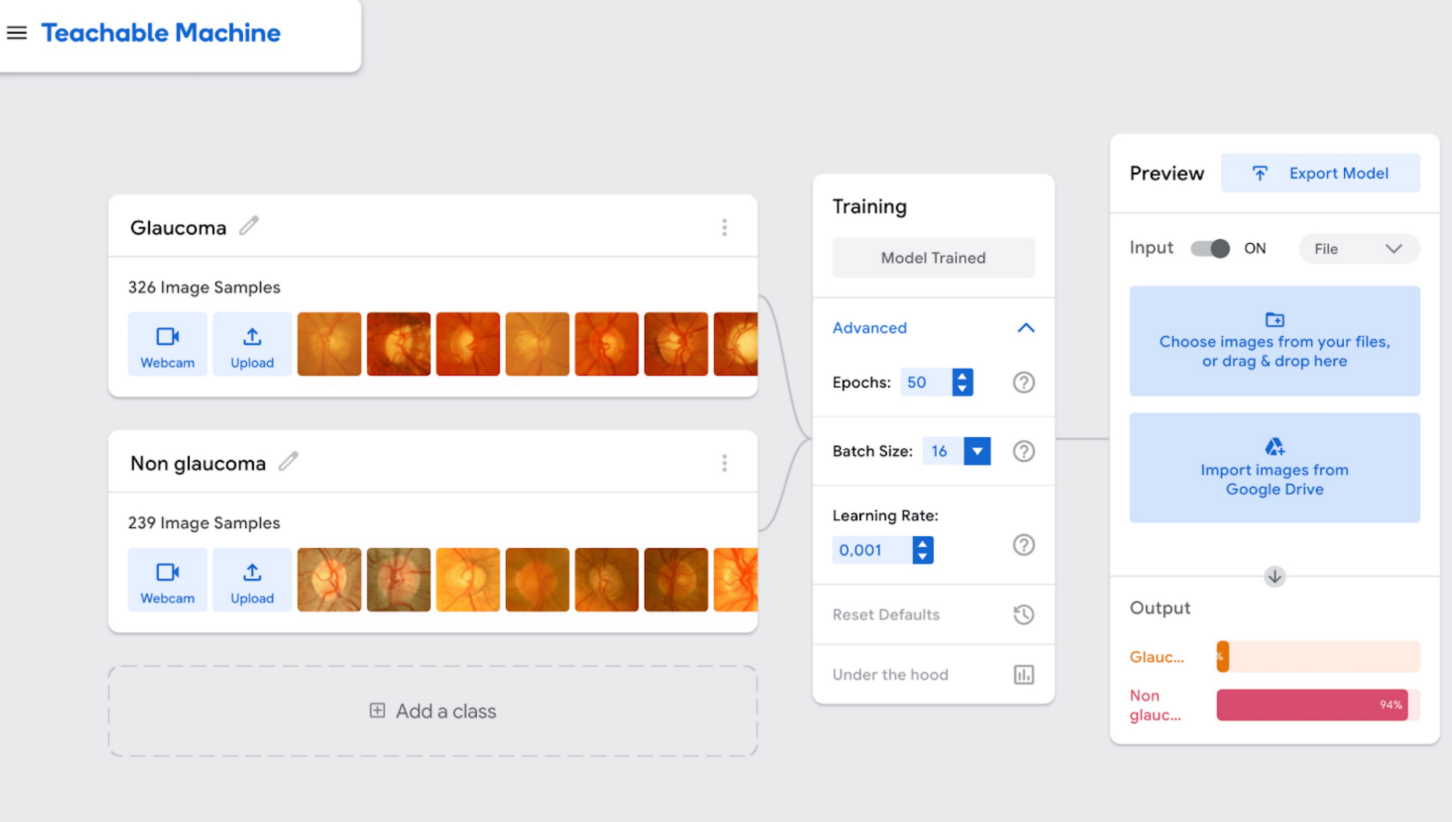
Teachable Machine platform

For Google's TM (version 2.0.7), which is based on the MobileNet-V2 architecture pre-trained on ImageNet, the model was trained for 50 epochs with a batch size of 16 and a fixed learning rate of 0.001 (Figure 1). The training was performed using Google Chrome (Google LLC, Mountain View, CA, USA) on a MacBook Air M1 (Apple Inc., Cupertino, CA, USA). During the testing phase, each test image was inputted manually, and classification results were recorded individually in a spreadsheet. The trained model is publicly accessible at https://teachablemachine.withgoogle.com/models/r60668V95/.

**Figure 2:**
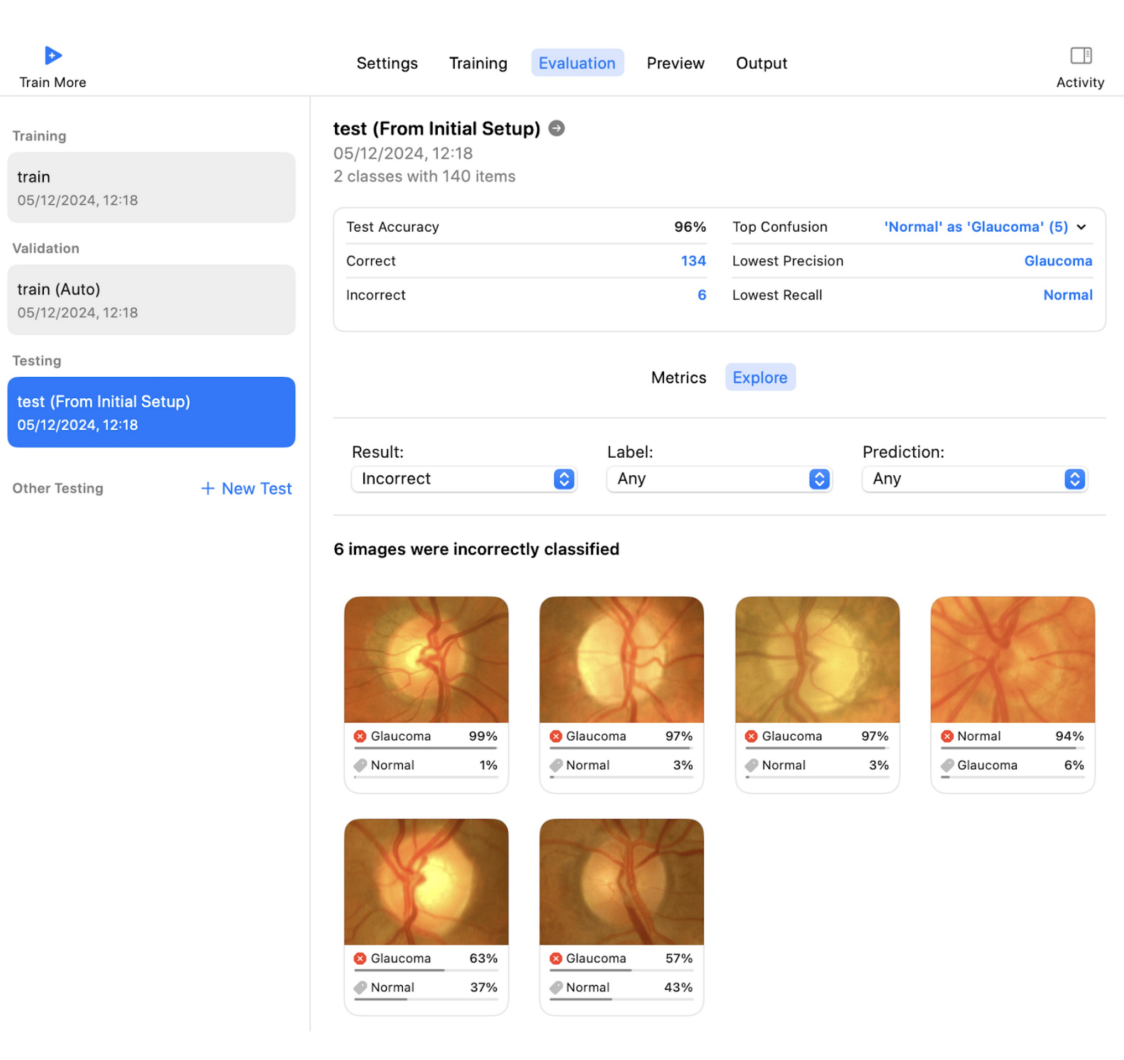
Create ML platform

For Apple's Create ML, input images were resized to 229 × 229 pixels, and the system automatically optimized the learning rate and batch size during training (Figure 2). The model was trained over 16 iterations on a MacBook Air M1, and the final trained model was exported in Core ML format. All 140 test images were input simultaneously, and classification results were automatically generated and recorded.

Statistical analysis 

Model performance was assessed using accuracy, sensitivity, specificity, F1 score, and Cohen's kappa coefficient. The validation dataset included 70 glaucomatous and 70 non-glaucomatous images. Accuracy was calculated as the proportion of correctly classified images. Sensitivity was defined as the proportion of true positives correctly identified. Specificity was defined as the proportion of true negatives correctly identified. The F1 score was computed as the harmonic mean of precision and sensitivity. Cohen's kappa coefficient was calculated to assess inter-rater agreement beyond chance. Confidence intervals (95%) were computed for each metric. 

A post hoc power analysis was conducted. The results of this analysis demonstrated a power of approximately 0.92, considering an alpha level of 0.05, an effect size of 0.5, and a two-tailed t-test. This suggests that the study possesses a substantial probability of detecting significant differences between the models, if they existed, which strengthens confidence in the validity of the presented results.

Statistical analyses were performed using IBM SPSS Statistics (IBM Corp., Armonk, NY) and R software (R Foundation for Statistical Computing, Vienna, Austria).

## Results

ResNet200d achieved superior performance in glaucoma classification. The model achieved a test accuracy of 99.29% and identified 70 true positive cases of glaucoma and 69 true negative cases of non-glaucoma, with zero false positives and one false negative. The area under the receiver operating characteristic curve (AUC) reached a perfect score of 1.00, indicating excellent discrimination between glaucomatous and non-glaucomatous cases across all classification thresholds. Additionally, the model attained an F1 score of 99.29%, reflecting a strong balance between precision and recall.

**Figure 3:**
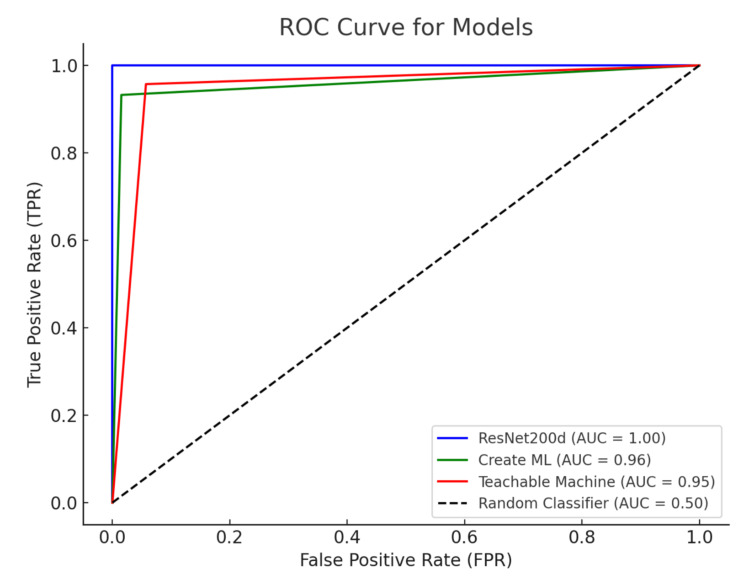
ROC curves for the Create ML, Teachable Machine, and ResNet200d models ROC: receiver operating characteristic; AUC: area under the receiver operating characteristic curve

The Create ML platform identified 69 true positive cases of glaucoma and 65 true negative cases of non-glaucoma, with one false positive and five false negatives observed. The F1 score was 95.83%. Create ML had an accuracy of 94.29%. The TM model identified 67 true positive cases of glaucoma and 66 true negative cases of non-glaucoma, with four false positives and three false negatives observed. The F1 score was 95.04%. TM had an accuracy of 95.04%. A summary of the performance metrics for all three models can be found in Table 1. The receiver operating characteristic (ROC) curves for all three models are presented in Figure 3.

**Table 1:** Model performance metrics PPV: positive predictive value; NPV: negative predictive value; AUC: area under the receiver operating characteristic curve

Model	Accuracy (%)	Sensitivity (%)	Specificity (%)	PPV (%)	NPV (%)	F1 score (%)	Cohen's kappa	AUC
ResNet200d	99.29 (95% CI: 98.03-100)	98.57 (95% CI: 92.34-99.75)	100.00 (95% CI: 94.80-100)	100.00 (95% CI: 94.73-100)	98.59 (95% CI: 92.44-99.75)	99.29 (95% CI: 96.47-100)	0.9857 (95% CI: 0.9567, 1.0000)	1.00
Create ML	94.29 (95% CI: 91.26-97.32)	93.24 (95% CI: 88.45-96.03)	98.48 (95% CI: 95.41-99.55)	98.57 (95% CI: 94.78-99.82)	92.86 (95% CI: 88.55-95.17)	95.83 (95% CI: 92.77-97.89)	0.92 (95% CI: 0.86-0.97)	0.96
Teachable Machine	95.04 (95% CI: 92.15-97.93)	95.71 (95% CI: 90.62-100)	94.29 (95% CI: 88.57-98.73)	94.37 (95% CI: 89.15-98.73)	95.65 (95% CI: 90.28-100)	95.04 (95% CI: 91.43-98.53)	0.90 (95% CI: 0.85-0.95)	0.95

## Discussion

This study has significant clinical relevance as it explores the potential of no-code AI platforms to democratize medical image analysis for glaucoma detection, particularly in resource-limited settings. The aim was to evaluate the performance of no-code AI platforms compared to traditional deep learning models, providing valuable insights for clinicians and researchers considering adopting these technologies. The traditional deep learning model (ResNet200d) outperformed models developed using no-code platforms (Google's TM and Apple's Create ML) in all evaluated metrics for classifying optic nerve fundus images into glaucoma and non-glaucoma categories. Despite the superior performance of ResNet200d, all models achieved high classification accuracy, confirming their applicability in medical image analysis.

Among the no-code models, the Create ML model exhibited a sensitivity of 93.24% and a specificity of 98.48%, minimizing false positives and suggesting suitability for confirmatory diagnostics. The model developed using TM demonstrated higher sensitivity (95.71%) but lower specificity (94.29%), making it more applicable for screening purposes. Both models showed strong agreement with expert classification, with Cohen's kappa values of 0.92 (Create ML) and 0.90 (TM). Create ML streamlined analysis by processing the entire dataset simultaneously and utilizing macOS Neural Engine optimizations. However, its reliance on Apple hardware limits accessibility. TM platform offers a highly accessible, web-based interface that does not require proprietary hardware. Its user-friendly design and compatibility with common browsers make it an attractive option for users seeking a flexible solution for model development. However, the individual input of images during testing added a level of manual effort that may be less practical for larger datasets.

When contextualizing these findings within the broader field, results align with prior studies evaluating the capabilities of code-free AI platforms. For example, Kirik et al. demonstrated that TM achieved 100% sensitivity and specificity in detecting vitreomacular interface diseases (VMIDs) in OCT images, with similarly high performance across individual disease subtypes [8]. Moreover, Korot et al. reported on the utility of multiple no-code platforms, including Create ML, for multi-modality medical image classification tasks. Their results indicated that Create ML models often performed slightly worse compared to platforms like Google AutoML, yet retained strong applicability for smaller datasets or where local computing is preferred [6]. In line with our findings, Balaskas et al. reported the use of Apple Create ML to distinguish between gradable and ungradable RetCam (Natus Medical Incorporated, Pleasanton, CA, USA) images in the context of retinopathy of prematurity (ROP) screening. Their model was trained on 1,732 images and validated on an independent dataset of 65 gradable and 40 ungradable images [9]. In the validation set, the model achieved a precision of 98% and a sensitivity of 100% for gradable image detection, highlighting its exceptional ability to identify usable images accurately [9]. However, in a testing set that better represented real-world conditions (with 1,430 gradable and 46 ungradable images), sensitivity dropped to 86% and specificity to 83%, reflecting the challenges of deploying such models in diverse clinical settings [9]. These findings illustrate the potential and limitations of Create ML and TM in classifying image quality for automated workflows.

From the literature, the current best-performing model for glaucoma diagnosis is a deep learning ensemble model combining Inception-v3 and ResNet-50, achieving an accuracy of 97.1% on the RIM-ONE dataset [6-9]. In comparison, the ResNet200d model in this study achieved an accuracy of 99.29% on the ACRIMA dataset, suggesting superior performance. However, direct comparison is limited due to the different datasets used. The results underscore the efficacy of traditional deep learning approaches in achieving high diagnostic accuracy, although they require more computational resources and technical expertise compared to no-code solutions. 

Despite the promising results, this study has limitations. The use of a single dataset (ACRIMA) limits the generalizability of the findings, as external validation on diverse datasets is required to confirm the robustness of these models across different populations and imaging conditions. Additionally, the dataset consisted exclusively of fundus images centered on the optic disc, without segmentation data, which may have reduced the models' ability to capture finer details of the optic disc and cup morphology. Furthermore, the prior imaging quality screening applied to the dataset may have introduced a selection bias, potentially overestimating the models' performance by excluding lower-quality images that are commonly encountered in real-world clinical settings. This selection bias could have led to an overestimation of the models' performance, as they were not tested on images with artifacts, noise, or poor contrast. Future studies should validate these models using larger and more heterogeneous datasets, incorporate segmentation-based features to enhance diagnostic accuracy, and evaluate their scalability and practical implementation in clinical workflows.

## Conclusions

While both Create ML and TM demonstrated strong performance in glaucoma classification, the traditional ResNet200d model outperformed both no-code platforms across all evaluated metrics, including accuracy, sensitivity, and specificity. Despite this, these findings highlight the potential of no-code machine learning platforms to advance medical image analysis and support clinical decision-making, particularly in resource-limited settings where access to advanced computational resources and expertise is limited. Future research should focus on enhancing these no-code solutions by integrating segmentation-based features and optimizing their performance on larger, more diverse datasets to improve their applicability in real-world clinical environments.
